# Chandipura virus after recurrent outbreaks in India: Five questions that matter for preparedness

**DOI:** 10.1371/journal.ppat.1014767

**Published:** 2026-11-06

**Authors:** Xue-Lian Zhang, Wei-Hao Tan, Jian-Wei Shao

**Affiliations:** School of Animal Science and Technology, Foshan University, Foshan, China; Washington University School of Medicine in Saint Louis: Washington University in St Louis School of Medicine, UNITED STATES OF AMERICA

## Introduction

Chandipura virus (CHPV), a sandfly-associated *vesiculovirus* in the family *Rhabdoviridae*, causes rapidly progressive and often fatal encephalitis in children [1,2]. It was first isolated in 1965 from patients with febrile illness in Maharashtra and emerged decades later as a cause of large-scale pediatric outbreaks in central and western India [1,3]. India experienced its largest CHPV outbreak in 20 years in 2024, when 245 acute encephalitis syndrome (AES) cases and 82 deaths were reported, including 64 laboratory-confirmed CHPV infections [4]. Only two years later, a preliminary snapshot as of 10 August 2026 recorded 25 deaths among 39 laboratory-confirmed cases in Gujarat [5]. These successive outbreaks highlight persistent biological uncertainties and gaps in preparedness. Clinical investigations document rapid progression from an initial febrile illness to seizures, altered consciousness, coma, or death [6,7]. Historical and field studies implicate phlebotomine sandflies [8–10], but the 2026 investigation had not yet identified a specific outbreak vector [5]. Here, we address five questions raised by these recurrences. We argue that preparedness should combine rapid pediatric triage and decentralized diagnosis with prospective clinical cohorts, hypothesis-driven vector and animal sampling, and comparative genomic analysis of CHPV sequences from human, arthropod, and animal samples to reconstruct transmission. The central challenge is not to rediscover CHPV during each outbreak, but to build the evidence and infrastructure needed between outbreaks.

### 1. Why can CHPV disease progress so rapidly in children?

The rapid clinical course is well documented, but its biological explanation remains incomplete. CHPV disease often begins with fever, vomiting, or diarrhea, then advances to seizures, altered consciousness, coma, and death over hours to days [3,6]. In the 2024 Gujarat investigation, most patients had an illness lasting only 24–72 hours, and multiorgan dysfunction was documented in severe cases [6]. A matched study included 54 children with laboratory-confirmed CHPV infection, with a median age of four years and 27 deaths, and the median interval from symptom onset to admission was only one day [7]. This compressed course allows minimal time for referral, diagnostic confirmation, or effective intervention after neurological decline has begun.

Cell-culture and animal models offer a partial explanation. Direct neurotropism, Fas-associated neuronal apoptosis, and microglial injury currently have the strongest mechanistic support, although mainly from experimental models [11,12]. Longitudinal clinical data from infected patients remain insufficient to distinguish the contributions of viral burden, neuroinflammation, systemic organ injury, and delays in care. The age distribution is also not fully explained. Underweight status and poor housing conditions were independently associated with confirmed disease in the 2024 matched analysis [7]. These associations do not establish whether nutrition alters susceptibility, disease severity, or exposure. Prospective cohorts should collect serial blood samples at predefined clinical time points, biomarkers of organ injury, treatment data, and outcomes, with cerebrospinal fluid sampling only when clinically indicated and safe. The clinically useful goal is a validated set of early warning markers that can trigger intensive monitoring before coma develops.

### 2. What do the 2024 and 2026 outbreaks actually tell us?

They demonstrate recurrent risk, but interpretation requires clearly distinguished denominators and geographic coverage. Between early June and 15 August 2024, India reported 245 AES cases and 82 deaths. Of these cases, 64 were laboratory-confirmed as CHPV [4]. The deaths were reported within the broader AES investigation and should not be divided by the smaller confirmed-case denominator. A subsequent laboratory investigation detected CHPV in 23.5% of tested encephalitis cases and reported a 47.4% case-fatality proportion among its defined CHPV group [6]. The matched field study reported 50% mortality among 54 confirmed children [7]. These estimates describe specific cohorts, not an infection-fatality risk for all community infections.

For 2026, the WHO South-East Asia Region Epidemiological Bulletin, 16th edition, published on 12 August 2026, cited media reporting of Gujarat State Health Department figures. As of 10 August, Gujarat had recorded 225 suspected cases since 30 June, including 39 laboratory-confirmed infections and 25 deaths among confirmed patients [5]. All reported cases were in children younger than 15 years. The bulletin also recorded intensified surveillance in Rajasthan and deployment of a National Joint Outbreak Response Team. These figures are a dated snapshot, not a final outbreak total.

Comparing years requires the same case definition, age range, geography, testing strategy, and reporting delay. Public health agencies should therefore label every figure as suspected, syndromic, tested, confirmed, or fatal, and attach a reporting date. This practice prevents invalid fatality calculations and makes surveillance trends interpretable.

### 3. Why does sandfly evidence not identify the vector in every outbreak?

The sandfly hypothesis is supported by more than ecological plausibility. CHPV has been isolated from field-collected *Sergentomyia* sandflies in Maharashtra and molecularly detected in *Sergentomyia* pools in Gujarat [8,9]. Experimental work demonstrated venereal transmission among *Phlebotomus papatasi* sandflies, providing a possible mechanism for maintaining CHPV within sandfly populations [10]. Together with the broader capacity of sandflies to transmit diverse viruses, these findings support sandfly surveillance and control [13]. They do not identify the dominant vector species in every outbreak, quantify its human-biting rate, or determine which vertebrates, if any, serve as amplifying or reservoir hosts.

The 2026 WHO bulletin described investigations of sandflies, mosquitoes, ticks, and mites and cautioned that attributing the outbreak to a specific vector was premature [5]. Approximately 70 samples from cattle, buffalo, and goats were also being assessed [5]. A practical CHPV-focused investigation should prioritize *Sergentomyia* species implicated by field detection and *Phlebotomus papatasi*, in which experimental venereal transmission has been demonstrated, while other arthropods are sampled in parallel. Investigators should conduct repeated collections at case households and epidemiologically comparable control locations, spanning relevant times and microhabitats, and document recent insecticide use. Negative viral RNA results would not exclude a vector if infection is rare or focal, sampling follows the transmission peak, or pooling reduces sensitivity. Conversely, a historical positive pool does not prove that the same species drives current transmission. Linking clinical, entomological, animal, and genomic evidence by place and time would make both positive and negative findings more informative.

### 4. How close are we to an antiviral or vaccine?

Clinical management remains supportive because no CHPV-specific antiviral is currently available [4]. Ribavirin inhibits replication in Vero cells [14]. Favipiravir reduced viral replication in vitro and protected neonatal mice in a lethal-challenge model [15]. Lycorine inhibited viral replication in cultured cells, apparently at an early replication step [16]. Time-resolved transcriptomics has also identified host-response pathways and repurposed compounds with activity in cultured cells [17]. Together, these findings justify standardized comparative preclinical testing, including toxicity, pharmacokinetics, and brain exposure, as well as therapeutic-window studies, before pediatric trial planning.

Vaccination has preclinical proof of concept. Recombinant CHPV glycoprotein induced neutralizing antibodies and protected mice, and a Vero cell-derived inactivated vaccine also protected immunized animals [18,19]. A recent analysis of 23 complete genomes collected from 1966–2024 suggested evolutionary constraint and strong purifying selection, although the small, geographically uneven dataset limits inference [20]. Genomic constraint does not establish antigenic conservation or cross-protection, which require functional evidence. Neither epitope predictions nor protection in mice establishes correlates of protection in children. These encouraging findings justify comparing candidate platforms, characterizing the breadth and durability of protection, assessing safety, and establishing scalable manufacturing before clinical evaluation across multiple outbreak seasons.

### 5. What would meaningful preparedness look like before the next outbreak?

Preparedness must operate on two distinct timescales (Fig 1). The clinical timescale is measured in hours. Community workers and front-line clinicians need clear criteria for urgent referral of a child with acute fever plus seizure or altered consciousness. Hospitals need rapid sampling, airway and seizure management, organ support, and access to intensive care. Decentralized reverse-transcription polymerase chain reaction (RT-PCR) testing or dependable referral networks should shorten the time to confirmation, but care must not be delayed while awaiting the result. Integrating CHPV testing into AES surveillance would also generate a denominator of tested patients rather than a collection of incomparable alerts [4–6].

**Fig 1:**
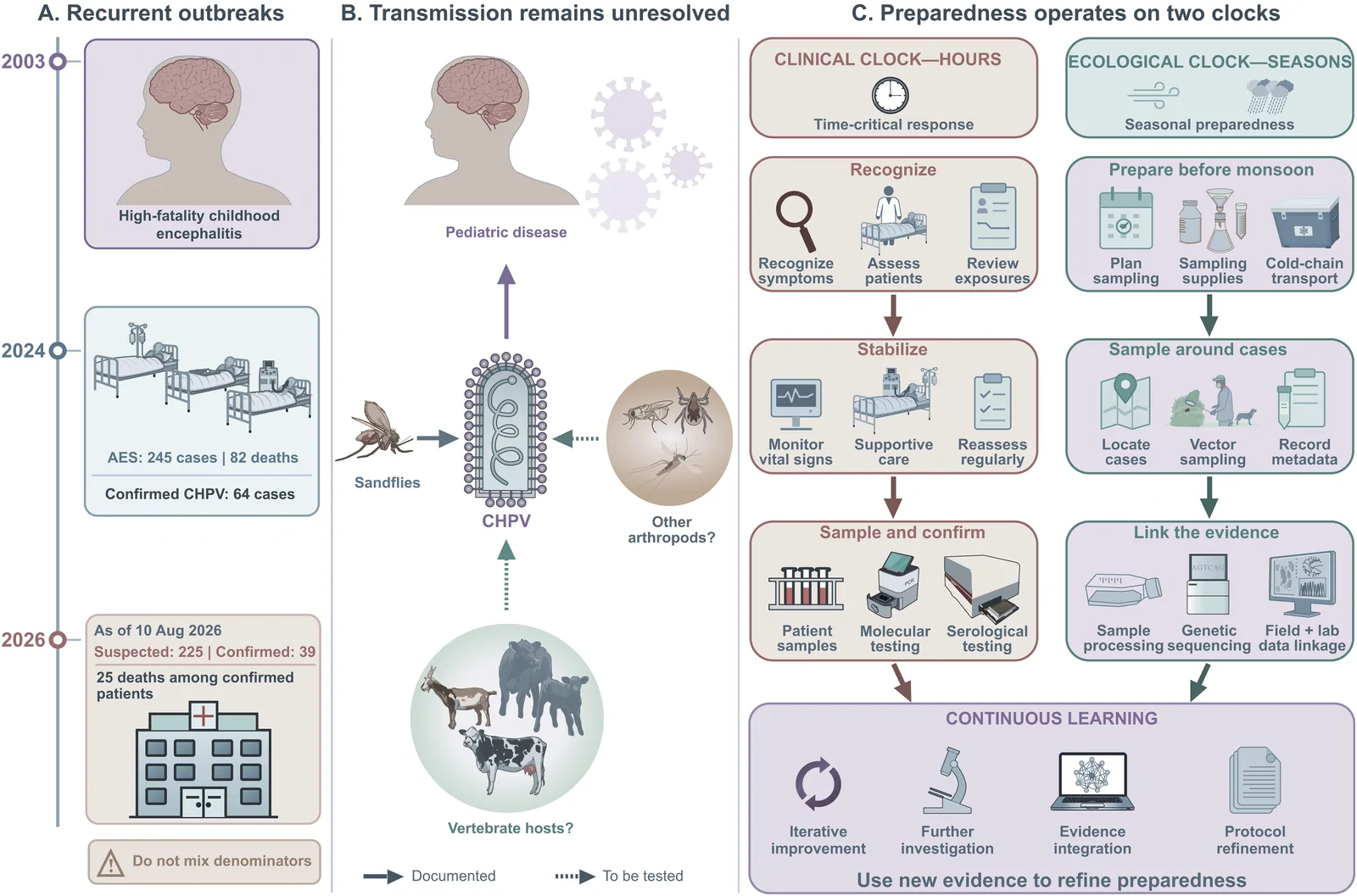
Recurrent Chandipura virus outbreaks, unresolved transmission ecology and preparedness on two timescales. **(A)** Recurrent outbreaks. Selected events in 2003, 2024 and 2026 highlight recurrent outbreaks associated with severe childhood encephalitis. Acute encephalitis syndrome (AES), suspected cases and laboratory-confirmed Chandipura virus (CHPV) infections represent distinct reporting categories; death counts must therefore be interpreted against their corresponding case definitions and denominators. The 2026 figures reflect the reporting date shown, 10 August 2026. The timeline is not drawn to scale. **(B)** Unresolved transmission ecology. The schematic distinguishes documented associations involving sandflies, CHPV and pediatric disease from potential roles of other arthropods and vertebrate hosts that require further investigation. Solid arrows indicate documented associations, whereas dashed arrows indicate relationships to be tested. The illustrated animals and arthropods represent candidate groups for investigation, not confirmed reservoirs or vectors in the outbreaks shown. **(C)** Preparedness on two timescales. The clinical clock operates over hours, emphasizing symptom recognition, patient assessment and exposure review; vital-sign monitoring, supportive care and regular reassessment; and patient sampling for molecular and serological testing. The ecological clock operates across seasons, emphasizing pre-monsoon sampling plans, supplies and cold-chain readiness; case-linked vector sampling and metadata collection; and integration of sample processing, genetic sequencing and linked field and laboratory data. Both pathways converge on continuous learning through further investigation, evidence integration and protocol refinement to inform subsequent preparedness.

The ecological timescale spans seasons, while investigations during an outbreak must still proceed promptly. The 2026 response linked human-health, veterinary, entomological, and laboratory agencies and incorporated active surveillance, serosurveys, animal sampling, and whole-genome sequencing [5]. We therefore prioritize three feasible actions before the next monsoon: establish rapid pediatric referral and supportive-care pathways; decentralize CHPV confirmation within AES surveillance; and pre-position protocols and supplies for case-linked vector sampling and sequencing. Clinical, vector, environmental, and animal data should be linked by place and time, with genomes paired to exposure and location metadata. Outbreak cohorts can then support validation of prognostic markers and preparation of countermeasure trials. These linked priorities would replace episodic responses with continuous learning between outbreaks.

## References

[1] Bhatt PN, Rodrigues FM. Chandipura: A new arbovirus isolated in India from patients with febrile illness. Indian J Med Res. 1967;55(12):1295–305. 4970067

[2] Walker PJ, Bejerman N, Blasdell KR, Debat H, Dietzgen RG, Fooks AR. ICTV virus taxonomy profile: *Rhabdoviridae*. J Gen Virol. 2026;107(6):002255. 10.1099/jgv.0.002255 PMC13249128 42262850

[3] Rao BL, Basu A, Wairagkar NS, Gore MM, Arankalle VA, Thakare JP. A large outbreak of acute encephalitis with high fatality rate in children in Andhra Pradesh, India, in 2003, associated with Chandipura virus. Lancet. 2004;364(9437):869–74. 10.1016/S0140-6736(04)16982-1 PMC7137741 15351194

[4] World Health Organization. Acute encephalitis syndrome due to Chandipura virus—India. Disease Outbreak News. 2024 [cited 2026 Sep 2]. Available from: https://www.who.int/emergencies/disease-outbreak-news/item/2024-DON529

[5] World Health Organization Regional Office for South-East Asia. WHO South-East Asia Region Epidemiological Bulletin. 16th ed. 12 August 2026; reporting period 27 July-9 August 2026 [cited 2026 Sep 2]. Available from: https://cdn.who.int/media/docs/default-source/searo/whe/wherepib/2026_16_searo_epi_bulletin.pdf

[6] Balachandran C, Chavan SP, Deoshatwar AR, Thankappan UP, Patil DR, Sawant PM, et al. Investigations into the outbreak of Chandipura virus encephalitis, Gujarat, India, 2024. J Med Virol. 2025;97(7):e70456. 10.1002/jmv.70456 40556437

[7] Swain AP, Varghese B, Balasubramanian S, Kanchan N, Dev AV, Mandriya K. Epidemiological investigation of Chandipura virus disease outbreak, Gujarat, India, July 2024: A matched case-control study. BMC Infect Dis. 2026;26(1):1491. 10.1186/s12879-026-13788-4 PMC13474709 42260362

[8] Sudeep AB, Bondre VP, Gurav YK, Gokhale MD, Sapkal GN, Mavale MS, et al. Isolation of Chandipura virus (*Vesiculovirus*: *Rhabdoviridae*) from Sergentomyia species of sandflies from Nagpur, Maharashtra, India. Indian J Med Res. 2014;139(5):769–72. 25027088 PMC4140043

[9] Damle RG, Sankararaman V, Bhide VS, Jadhav VK, Walimbe AM, Gokhale MD. Molecular Evidence of Chandipura Virus from *Sergentomyia* species of Sandflies in Gujarat, India. Jpn J Infect Dis. 2018;71(3):247–9. doi: 10.7883/yoken.JJID.2017.397 29709979

[10] Mavale MS, Fulmali PV, Geevarghese G, Arankalle VA, Ghodke YS, Kanojia PC, et al. Venereal transmission of Chandipura virus by *Phlebotomus papatasi* (Scopoli). Am J Trop Med Hyg. 2006;75(6):1151–2. doi: 10.4269/ajtmh.2006.75.1151 17172384

[11] Ghosh S, Dutta K, Basu A. Chandipura virus induces neuronal death through Fas-mediated extrinsic apoptotic pathway. J Virol. 2013;87(22):12398–406. doi: 10.1128/JVI.01864-13 24027318 PMC3807914

[12] Verma AK, Ghosh S, Pradhan S, Basu A. Microglial activation induces neuronal death in Chandipura virus infection. Sci Rep. 2016;6:22544. doi: 10.1038/srep22544 26931456 PMC4773833

[13] Jancarova M, Polanska N, Volf P, Dvorak V. The role of sand flies as vectors of viruses other than phleboviruses. J Gen Virol. 2023;104(4):10.1099/jgv.0.001837. doi: 10.1099/jgv.0.001837 37018120

[14] Balakrishnan A, Mun AB. Ribavirin inhibits Chandipura virus replication in Vero cells. J Med Virol. 2020;92(12):2969–75. doi: 10.1002/jmv.26184 32543712

[15] Pavitrakar DV, Bondre VP. Antiviral effect of Favipiravir against Chandipura virus in vitro and in vivo. J Med Virol. 2023;95(6):e28840. doi: 10.1002/jmv.28840 37254846

[16] Agrawal T, Singh S, Kaur G, Rathore S, Sharma H, Rajput G, et al. Lycorine inhibits Chandipura virus replication in vitro. J Med Virol. 2025;97(10):e70636. doi: 10.1002/jmv.70636 41045067

[17] Jhala D, Shah P, Shah D, Raval I, Puvar A, Bagatharia S, et al. Time-resolved transcriptomic profiling of Chandipura virus infection reveals dynamic host responses and host-directed therapeutic targets. Int J Mol Sci. 2026;27(8):3364. doi: 10.3390/ijms27083364 42074008 PMC13115980

[18] Venkateswarlu CH, Arankalle VA. Recombinant glycoprotein based vaccine for Chandipura virus infection. Vaccine. 2009;27(21):2845–50. doi: 10.1016/j.vaccine.2009.02.089 19428894

[19] Jadi RS, Sudeep AB, Barde PV, Arankalle VA, Mishra AC. Development of an inactivated candidate vaccine against Chandipura virus (*Rhabdoviridae*: *Vesiculovirus*). Vaccine. 2011;29(28):4613–7. doi: 10.1016/j.vaccine.2011.04.063 21549791 PMC7115669

[20] Kuri PR, Sharma A. Unravelling the genomic epidemiology and antigenic evolution of Chandipura virus: A genetically constrained neurotropic threat. PLoS Negl Trop Dis. 2026;20(8):e0014565. doi: 10.1371/journal.pntd.0014565 42546036 PMC13489527

